# CO_2_ in Lyotropic Liquid Crystals: Phase Equilibria Behavior and Rheology

**DOI:** 10.3390/polym11020309

**Published:** 2019-02-12

**Authors:** Sandra Rodríguez-Fabià, Jens Norrman, Hanna K. Knuutila, Johan Sjöblom, Kristofer Paso

**Affiliations:** Department of Chemical Engineering, Norwegian University of Science and Technology (NTNU), 7491 Trondheim, Norway; jens.norrman@ntnu.no (J.N.); hanna.knuutila@ntnu.no (H.K.K.); johan.sjoblom@ntnu.no (J.S.); kristofer.g.paso@ntnu.no (K.P.)

**Keywords:** Lyotropic liquid crystals, CO_2_ capture, phase behavior, PEO-PPO-PEO, MEA, CO_2_ absorption, rheology of liquid crystals

## Abstract

The CO_2_ absorption of liquid crystalline phases of poly(ethylene oxide)-poly(propylene oxide)-poly(ethylene oxide) (Pluronic L92, (EO)_8_(PO)_47_(EO)_8_), monoethanolamine (MEA), and water, with a composition of 60% L92/10% MEA/30% water has been investigated to assess potential use in carbon capture and storage applications. Vapor–liquid equilibrium data of the liquid crystalline system with CO_2_ was recorded up to a CO_2_ partial pressure of 6 bar, where a loading of 38.6 g CO_2_/kg sample was obtained. Moreover, the phase transitions occurring during the loading process were investigated by small angle X-ray scattering (SAXS), presenting a transition from lamellar + hexagonal phase to hexagonal (at 25 °C). In addition, the rheology of samples with varying loadings was also studied, showing that the viscosity increases with increasing CO_2_-loading until the phase transition to hexagonal phase is completed. Finally, thermal stability experiments were performed, and revealed that L92 does not contribute to MEA degradation.

## 1. Introduction

Triblock copolymers consisting of poly(ethylene oxide)-poly(propylene oxide)-poly(ethylene oxide) (PEO-PPO-PEO) are used extensively in industry. These amphiphilic copolymers are also known by the trade names Pluronic or Poloxamer. Among the wide range of applications of PEO-PPO-PEO copolymers, the most common fields are detergency, foaming, emulsification, lubrication, dispersion stabilization, and drug delivery [1,2,3,4,5,6]. 

Properties of PEO-PPO-PEO copolymers have been studied in some detail. Modification of the hydrophilic/hydrophobic balance of these polymers gives rise to the formation of numerous microstructures [7]. In water, PEO-PPO-PEO copolymers self-assemble and form liquid crystalline structures, including both normal and reverse phases [8,9,10]. The interfacial curvature of these microstructures can be tuned via the addition of a cosolvent, due to dissimilar swelling of the polymer blocks with selective solvents [11,12,13]. In addition to the use of cosolvents with varying polarities, the phase behavior of PEO-PPO-PEO copolymers can also be modified by addition of various cosolutes to the system. For example, the increased polarity of water with respect to the polymer due to the addition of salts favors phase separation [14,15]. 

Furthermore, rheological properties of liquid crystalline phases have been investigated. Lamellar phases formed by low molecular weight surfactants exhibit shear thinning behavior [16]. Mezzenga et al. studied the rheological behavior of several lamellar, hexagonal, and bicontinuous cubic phases formed by lipids in water [17]. In their work, they showed that each liquid crystalline phase has a characteristic viscoelastic behavior. For instance, they inferred that the elastic behavior of lamellar phases is caused by the slippage of the lamellae, whereas hexagonal phases have a viscoelastic nature [17]. Regarding PEO-PPO-PEO copolymers in aqueous systems, there have been several studies relating polymer microstructure to rheological behavior [18,19]. Aqueous solutions of Pluronic F127 below 12.5 wt % display Newtonian behavior because the polymer micelles are well separated in solution. Above 17 wt % F127, the system shows gel-like behavior due to interactions of large aggregates formed by cubic packing of the polymer micelles [18]. In addition, the viscoelastic behavior of F127 solutions was investigated by Hyun et al. [19]. They studied large amplitude oscillatory shear (LAOS) behavior of the samples by performing strain and frequency sweeps. According to rheological behavior outside the linear viscoelastic regime, complex fluids can be classified in four categories: type I (strain thinning), type II (strain hardening), type III (weak strain overshoot), and type IV (strong strain overshoot) [20]. Type I and type II describe opposing behaviors: in type I, G’, and G’’ decrease with increasing strain, whereas in type II they increase. In type III G’ decreases, whereas G’’ first increases and then decreases, and in type IV both G’ and G’’ undergo an overshoot [20]. The LAOS behavior of Pluronic samples depends on the microstructure of the samples, which undergo sol-gel transitions triggered by temperature changes [19]. 

In a previous publication, we described the phase behavior of Pluronic L92/water/monoethanolamine (MEA) systems as potential solvents for carbon capture and storage (CCS) [21]. MEA is an extensively studied solvent for post-combustion CO_2_ capture by chemical absorption [22,23,24]. The reaction between MEA and CO_2_ occurs through the formation of a zwitterion, and the successive formation of a carbamate [24,25]. PEO-PPO-PEO copolymers are not utilized industrially for CO_2_ capture; however, PEO derivatives are used for physical absorption of CO_2_ at low temperature and elevated pressure conditions, and are commercially available under the trade names Selexol and Genosorb [26,27,28]. 

The liquid crystal concept investigated in this publication is an integrated technology that in contrast with traditional solvents for CO_2_, which are only used for capture, includes capture of CO_2_, transport and storage. Polymeric liquid crystals provide an increased density of functionalities that can interact favorably with CO_2_. Moreover, liquid crystals supply an additional sealing mechanism to the traditional underground storage conditions. The current publication is a continuation of our previous work [21]. Based on our previous results, we selected liquid crystals with an optimized composition for capturing CO_2_, that is, the composition with the highest MEA concentration. Here, we select a liquid crystalline phase consisting of 60% L92/10% MEA in water and we study the maximum CO_2_-loading that is achievable at 6 bar pressure. In addition, we also investigate phase transitions occurring during the loading process in a separate experiment. The phase behavior of the samples taken during the reaction with CO_2_ is investigated using small angle X-ray scattering (SAXS), and the rheological behavior of the samples is also characterized. Moreover, thermal degradation experiments of CO_2_-loaded samples are performed to assess if L92 influences the degradation of MEA.

## 2. Materials and Methods 

### 2.1. Materials

Pluronic L92 (poly(ethylene oxide)-*block*-poly(propylene oxide)-*block*-poly(ethylene oxide) (PE 9200, *M*_n_ ≈ 3650 g/mol, 20 wt % PEO) was provided by BASF Corporation (Ludwigshafen, Germany). The composition of Pluronic L92 can be represented as (EO)_8_(PO)_47_(EO)_8_ [6,8]. Monoethanolamine (MEA, ≥99.0%) and barium chloride dehydrate (≥99.0%) were purchased from Sigma Aldrich (Saint Louis, MO, USA). Carbon dioxide (gas, 99.9%) was purchased from AGA, NaOH 0.1 N, HCl 0.1 N, and H_2_SO_4_ 0.1 N were purchased from Merck KGaA (Darmstadt, Germany). All chemicals were used as received. Milli-Q water was used as solvent (18.2 MΩ cm). Compositions of liquid crystalline samples and MEA solutions are expressed in weight %.

### 2.2. Vapor–Liquid Equilibrium (VLE) Experiments

60% L92/10% MEA was prepared by weighing corresponding amounts of polymer, MEA, and Milli-Q water into a beaker and stirring the mixture with a glass rod. A portion of the sample was retained in order to perform further tests. The remaining mixture was transferred into a glass reactor, and used to perform the VLE experiments. 

60% L92/10% MEA was loaded with CO_2_ in a CPA202 reaction calorimeter (ChemiSens AB, Lund, Sweden). Detailed information about the calorimeter setup is described by Hartono et al. [29]. The reactor was submersed in a CPA202BU thermostat, using diethylene glycol as heating medium. The calorimeter is a glass reactor with a stainless steel lid and a total volume of 258.8 ± 0.2 cm^3^. The reactor is equipped with a Pt-100 temperature sensor (accuracy 0.1 °C), a SENSIT pressure gauge (1–10 Bar, accuracy 0.15% FS), and a propeller stirrer. All operational parameters (e.g., reactor temperature and pressure, CO_2_ temperature and pressure, heat flow, CO_2_ flow, etc.) were recorded as function of time using ChemiCall software from Chemisens. CO_2_ was fed to the reactor via a mass flow controller (0.5 nL/min, Bronkhorst^®^ Hightech, Ruurlo, Netherlands). 

The experiment was conducted at 30 °C, and approximately 110 g of sample was used. Vacuum was applied to the reactor prior to CO_2_ injection in order to remove air. CO_2_ was added batch-wise for 10 seconds in several injections until the pressure in the reactor reached 6 bar. After each injection, the system was allowed to equilibrate until predetermined equilibrium conditions were reached, and the system remained within the predetermined conditions range for 10 min. Stability conditions were defined as ΔP = ± 5 mbar, and ΔT = ± 0.05 °C. The experiment was programmed to end 8 h after equilibrium conditions of the last injection were attained. Stirring of the sample was interrupted during the experiment due to the high viscosity the sample attained after several CO_2_ injections. 

The reaction mechanism between MEA and CO_2_ is described as follows [25]: first, MEA (here denoted as RNH_2_) reacts with a CO_2_ molecule, forming a zwitterion, as shown in Equation (1).

(1) $${\mathrm{CO}}_{2}+{\mathrm{RNH}}_{2}⇄{\mathrm{RNH}}_{2}{}^{+}{\mathrm{COO}}^{-}$$

In the next step of the reaction, a base (B) present in the system deprotonates the zwitterion, forming a carbamate (Equation (2)):

(2) $${\mathrm{RNH}}_{2}{}^{+}{\mathrm{COO}}^{-}+B⇄{\mathrm{RNHCOO}}^{-}+{\mathrm{BH}}^{+}$$

The base (B) is typically water, OH^−^ ions, or MEA. In the case of MEA, deprotonation of the carbamate is expressed as:

(3) $${\mathrm{RNH}}_{2}{}^{+}{\mathrm{COO}}^{-}+{\mathrm{RNH}}_{2}⇄{\mathrm{RNHCOO}}^{-}+{\mathrm{RNH}}_{3}{}^{+}$$

The overall reaction between MEA and CO_2_ is shown in equation (4):

(4) $${\mathrm{CO}}_{2}+2{\mathrm{RNH}}_{2}⇄{\mathrm{RNHCOO}}^{-}+{\mathrm{RNH}}_{3}{}^{+}$$

### 2.3. CO_2_ Absorption of 60% L92/10% MEA in Round Bottom Flask

In order to obtain samples at various stages of the absorption process, and investigate the phase transitions, CO_2_ loading of 60% L92/10% MEA was performed in a 250 mL three-necked round bottom flask equipped with a condenser to prevent solvent evaporation. Corresponding amounts of polymer, MEA, and Milli-Q water were added to the round bottom flask. 60% L92/10% MEA was loaded by bubbling CO_2_ from two of the flask’s inlets, and was stirred manually. The reaction was performed at room temperature over the course of several days. Samples were taken before and during the absorption process until the loading of 60% L92/10% MEA with CO_2_ was completed. The CO_2_ loading of the samples was determined by titration methods described in Section 2.9. 

### 2.4. Thermal Degradation Experiments

2.5 g of CO_2_-loaded 60% L92/10% MEA were put into a glass test tube, and then into a 316 stainless steel cylinder closed with a Swagelok^®^ cap, as described by Fytianos et al. [30]. The objective was to see if L92 will influence the degradation of MEA negatively. Unfortunately, it was not possible to run the experiments at temperatures typically used in the literature (120–150 °C) [30] due to L92 not being stable at such high temperatures. Six parallels were prepared, and they were placed in a heating oven maintained at 80 °C. Three parallels were removed from the oven after 1 week, and the remaining three, after 7 weeks. The cylinders were weighted before and after the experiment to detect any mass losses caused by leakage. The total alkalinity of the samples was determined before and after the experiments using titration methods described in Section 2.9. 

### 2.5. Thermogravimetric Analysis (TGA)

TGA experiments were performed using a Q600 SDT Thermogravimetric Analyzer with DSC (TA Instruments, New Castle, DE, USA). The sensitivity of the microbalance was 0.1 μg. The samples were maintained at isothermal conditions for 0.5 min, and then they were heated at 10 °C/min to 450 °C. Sample weight loss and rate weight loss were recorded as functions of time and temperature. TGA experiments were performed at atmospheric pressure, under a nitrogen atmosphere at a flow rate of 100 mL/min. 

### 2.6. Cross-Polarized Visual Inspection

All samples were inspected between cross-polarizers to check for birefringence. Isotropic phases (such as cubic phases and liquids with no long-range organization) are not birefringent when they are observed through polarized light, while anisotropic phases (such as lamellar and hexagonal phases) are birefringent when they are observed through cross-polarizers. Observation through crossed polarizers is a simple, yet effective, way of differentiating between different possible phases in a sample.

### 2.7. Small Angle X-ray Scattering (SAXS)

In order to establish the liquid crystalline phases and associated lattice parameters, SAXS experiments were performed on a Bruker Nanostar SAXS system equipped with a Våntec-2000 detector (Bruker AXS GmbH, Karlsruhe, Germany). Κα radiation (λ = 1.524 Å) was provided by a IμS Cu microsource (Incoatec, Geestacht, Germany) operating at 50 kV and 60 mA. The samples were placed in a sandwich cell with Kapton windows, and measurements were performed at 15, 25, 35, and 45 °C. The spectrum of the sample loaded in the calorimeter was only measured at 25 °C. The raw scattering data was calibrated to absolute intensity scale using water as standard. The 1D scattering profile as a function of the scattering vector was obtained by radially averaging the scattering data. The scattering of the empty cell was subtracted from the corresponding measured sample. 

The relative positions of the Bragg diffraction peaks were used to determine the structure of the lyotropic liquid crystalline phases [8,9]. The positions of the peaks of the lamellar and the hexagonal phases follow the relationships 1:2:3:4… and 1:$\sqrt{3}$:2:$\sqrt{7}$:3…, respectively. The lattice parameters of the lamellar (5) and hexagonal (6) phases can be calculated from the position of the most intense diffraction peak (*q*_1_):

(5) $$q_{1}=\frac{2\pi }{d}$$

(6) $$q_{1}=\frac{4\pi }{a\sqrt{3}}$$

where *d* is the lamellar periodicity, *a* is the distance between centers of adjacent cylinders.

### 2.8. Rheology

Rheological measurements were performed using a Physica MCR 301 rheometer (Anton Paar, Graz, Austria). Sandblasted cone-and-plate geometry with cone angle was 2.009° and radius 39.975 mm was used. The viscosity was measured as a function of the shear rate. The range of shear rate was 0.01 to 100 s^−1^. Oscillatory measurements were performed to determine viscoelastic behavior in the linear viscoelastic regime. The elastic modulus (G’) and the loss modulus (G’’) as a function of strain amplitude were measured at a frequency of 1 Hz and the strain amplitude range was 0.1 to 100%. Frequency sweep of the elastic modulus (G’) and the loss modulus (G’’) was made at a strain amplitude of 0.1% with a frequency range of 0.1 to 100 Hz. All measurements were performed at 25 °C. In addition, the viscosity of unloaded 60% L92/10% MEA was measured at 15 °C.

### 2.9. Titration Methods

The CO_2_ loading and the amine concentration of 60% L92/10% were determined by titration. The absorbed CO_2_ was determined following the procedure described by Weiland et al. [31]. A loaded sample weighing 0.6 g was added to 25 mL of 1 N BaCl_2_ and 50 ml of 0.1 N NaOH in an Erlenmeyer flask. The mixture was stirred until the sample was fully dissolved. The flask was covered with a stopper equipped with a vapor tube, and was heated until the reaction mixture started boiling. A BaCO_3_ white precipitate was formed. After 4 min of boiling, the heating was stopped and the flask was cooled down. The solution was filtrated using a 0.45 μm HA MF-Millipore membrane filter. The precipitate was transferred to a beaker and dissolved in 50 ml of deionized water and 40 mL of 0.1 N HCl solution. After BaCO_3_ was completely dissolved, the sample was titrated with 0.1 N NaOH in a Titrando autotitrator (Metrohm, Herisau, Switzerland). Following the same procedure, a blank sample was prepared to account for CO_2_ present in air. In this case, 10 mL of HCl was added prior to the titration. The concentration of amine groups was determined by alkaline titration according to the procedure described by Ma’mun et al. [32]. A sample weighing 0.2 g was dissolved in 50 mL of deionized water, and the sample was titrated with 0.1 M H_2_SO_4_ in a G20 Compact titrator (Mettler Toledo, Oslo, Norway).

## 3. Results and Discussion

### 3.1. VLE Experiments

**Figure 1:**
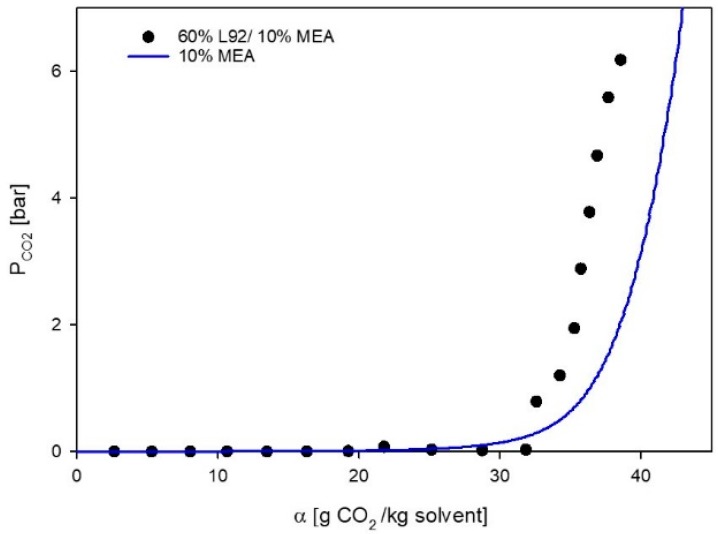
Vapor–liquid equilibrium (VLE) data for 60% L92/10% MEA, and 10% monoethanolamine (MEA). ⬤ Experimental data, this work; solid line, CO_2_ pressure above aqueous 10% solution as a function of CO_2_ loading at 30 °C [33].

The CO_2_ absorption of a liquid crystalline sample containing MEA (60% L92/10% MEA) was measured, and the VLE data is shown in Figure 1, were the partial pressure of CO_2_ in the reactor (P_CO2_) has been plotted as a function of loading (α). The final loading of the sample is 38.6 g CO_2_/kg sample. The VLE data of 60% L92/10% MEA shows that the sample exhibits dual behavior: first chemical absorption takes place and then physical absorption occurs. After each of the first 11 injections, the pressure in the reactor drops to approximately 0 bar, indicating that all CO_2_ has reacted with MEA. Hence, the loading of the solution increases, while the partial pressure of CO_2_ in the reactor remains low. After loading 32 CO_2_/kg sample, the partial pressure in the reactor starts to increase fast and linearly, indicating that physical absorption is occurring [27]. In Figure 1, together with experimental data, the vapor–liquid equilibria model for 10% MEA has been plotted at 30 °C [33]. The behavior of the two systems (both containing 10% MEA) is very similar. This indicates that the absorption of CO_2_ into the developed liquid crystal (g CO_2_/kg sample) is limited by the amount of amine, and that all the amine in the liquid crystal is still active and can be used to absorb CO_2_. However, due to the high viscosity of the unloaded and loaded liquid crystal (see Section 3.3.1), the VLE experiments took much longer than in typical aqueous MEA solutions. 

During the experiment a change in phase behavior of 60% L92/10% MEA was observed, where the physical properties of the sample changed. In order to investigate this phase transition, loading of the sample was performed in a round bottom flask.

### 3.2. Phase Behavior of 60% L92/10% MEA during CO_2_ Absorption 

Pure CO_2_ was bubbled through 60% L92/10% MEA in a round bottom flask in order to investigate the phase transition occurring during the loading process. Samples were acquired at various arbitrary times during the experiment and analyzed by TGA to check if there was water loss due to CO_2_ bubbling. The CO_2_-loading of the samples was quantified by titration. In this manuscript, the samples are named according to the CO_2_-loading, α (Table 1).

**Figure 2:**
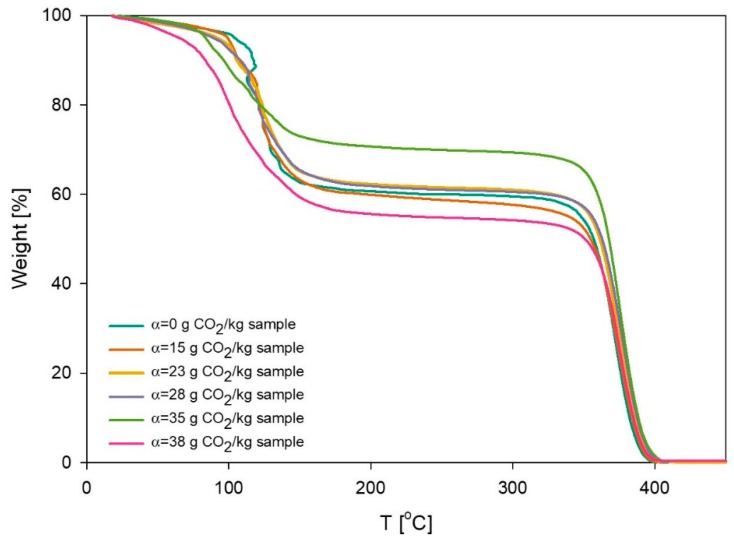
Change of weight percentage of the sample 60% L92/10% MEA as a function of temperature. Samples were taken at different points in time during the loading experiment.

Results of the TGA measurements are shown in Figure 2, and the initial (unloaded) sample was used as a control reference. It is evident from the plot that 40% of the weight is lost between 20 °C and 150 °C. This corresponds to loss of water and MEA. Subsequently, the curve reaches a plateau, followed by a decrease around 350 °C. This behavior corresponds to the polymeric portion of the sample, which starts to decompose above 350 °C. The TGA results confirm that the initial sample consisted of 39% solvents and 61% of polymer. The results showed that until the sample reached a loading of 27 g CO_2_/kg sample, the composition did not change. The polymer concentration of the sample with loading 35 g CO_2_/kg sample reached 71%, due to evaporation of water caused by bubbling of CO_2_. Therefore, the composition of this sample was adjusted by adding Milli-Q water to the reaction mixture. The loading experiment was considered to be complete when the loading of the sample reached 38 g CO_2_/kg sample. TGA results showed that the final sample composition consisted of 57% polymer and 43% solvent. 

The CO_2_-loading of the calorimeter sample at one bar is approximately 34 g CO_2_/kg sample, whereas the final loading obtained in the round-bottom flask experiment is 38 g CO_2_/kg sample. The 10% difference between these two values might be caused by slight variations in the composition of the samples, as well as the different techniques used to determine the loading of the samples. The loadings obtained for this liquid crystalline system at 1 bar, are slightly higher than the values for three different thermotropic liquid crystals reported by Chen et al. (MBBA, PCH5, and PCH8-CNS), where the highest loadings they measured were between 7–8 mg CO_2_/g liquid crystal [34].

**Figure 3:**
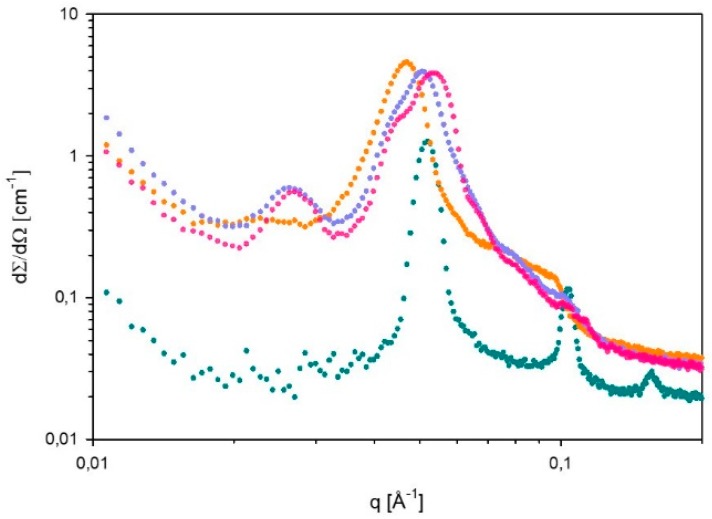
SAXS spectrum of 60% L92/10% MEA with various degrees of loading (⬤α = 0, ⬤α = 15, ⬤α = 27, and ⬤α = 38 g CO_2_/kg sample), recorded at 15 °C.

**Figure 4:**
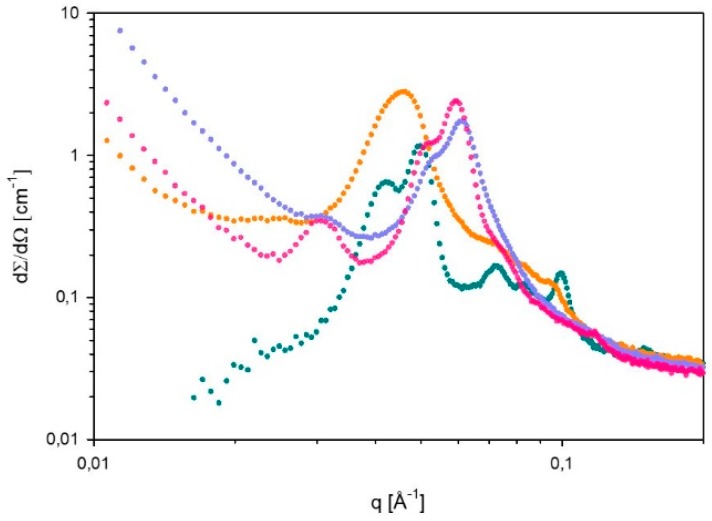
Small angle X-ray scattering (SAXS) spectrum of 60% L92/10% MEA with various degrees of loading (⬤α = 0, ⬤α = 15, ⬤α = 27, and ⬤α = 38 g CO_2_/kg sample), recorded at 25 °C.

SAXS spectra of 60% L92/10% MEA with loadings α = 0, 15, 27, and 38 g CO_2_/kg sample were recorded at 15, 25, 35, and 45 °C. However, it should be noted that it was not possible to distinguish between hexagonal and reverse hexagonal phases. Therefore, the symbol used for hexagonal phases (H) represents both normal hexagonal and reverse hexagonal phases. The spectra measured at 35 and 45 °C showed that the microstructure of the samples was destroyed, and therefore these spectra are not presented. The results for unloaded 60% L92/10% MEA (α = 0 g CO_2_/kg sample) were previously reported [21]. The SAXS spectra displayed in Figure 3 and Figure 4, show how the microstructure of the sample changes as the CO_2_-loading increases at 15 and 25 °C, respectively. In addition, the corresponding lattice parameters have been calculated, and are shown in Table 2. 

The SAXS spectrum recorded at 15 °C (Figure 3) shows that the unloaded sample forms a lamellar phase (Lα) [21]. At α = 15 g CO_2_/kg sample, a broad intense peak forms, with some shoulders at higher q values. However, the relative peak positions do not follow the pattern of any liquid crystalline structure. The broad peak at α = 15 g CO_2_/kg sample is slightly shifted to lower q values in comparison with the most intense peak at α = 0, implying that the distance between aggregates increases. When the loading reaches 27 g CO_2_/kg sample, a new Bragg peak appears at low q values, and the most intense peak in this spectrum has a shoulder. The ratio between the three peaks follows the relationship established for hexagonal phases. In this case, the peaks are shifted towards higher q values, meaning that the aggregates are more compressed than the ones with loadings 0 and α = 15 g CO_2_/kg sample. Similarly, the spectrum of the sample with α = 38 g CO_2_/kg sample follows the same trends as the sample with α = 27 g CO_2_/kg sample. In this case, there is a clearer distinction between the two most intense peaks that are merged. The relationship between the less intense peak at low q values and the two intense peaks is 1÷$\sqrt{3}$÷2, which corresponds to a hexagonal phase.

At 25 °C (Figure 4), when there is no CO_2_ present, the sample has a defined microstructure consisting of coexisting lamellar and hexagonal phases [21]. In the case of the other samples, the phase behavior is analogous at 15 and 25 °C. As the degree of CO_2_-loading increases, the microstructure shifts towards hexagonal phase. Comparing the samples with loadings 0 and 15 g CO_2_/kg sample, it can be seen that a broad intense peak appears, which seems to be the result of the combination of the most intense peaks of the lamellar and hexagonal phases present in the unloaded sample, and suggests the presence of amorphous aggregates. As discussed for 15 °C, the main peak of α = 15 g CO_2_/kg sample is slightly shifted to lower q values with respect to the intense peaks of the unloaded sample, indicating the larger distance between aggregates. Moreover, there are shoulders in the spectrum α = 15 g CO_2_/kg sample in the positions where the Bragg peaks of the unloaded sample appear, indicating that part of the structure is still remaining. At higher q values two shoulders appear which follow the relationship 1÷$\sqrt{3}$÷2 with respect to the most intense peak, possibly indicating the presence of remaining hexagonal aggregates. The lattice parameters of this sample are not calculated due to its undefined microstructure.

The spectra obtained for loadings of 27 and 38 g CO_2_/kg sample follow the same behavior as described for 15 °C. In both cases, a small Bragg peak starts to appear at low q values. The spectra of both samples show the presence of an intense peak with a shoulder, which seems to correspond to two merged peaks. The distinction between these peaks is clearer at 25 °C than at 15 °C, and it is more defined for the highest loading, suggesting that the aggregates are more ordered and that at 15 °C the transition towards hexagonal phase is still ongoing when the loading is 27 g CO_2_/kg sample. At 25 °C, the relationship between the peaks at 27 and 38 g CO_2_/kg sample follows the ratio 1÷$\sqrt{3}$÷2, which corresponds to a hexagonal phase. However, the positions of the main peaks of α = 27 and 38 g CO_2_/kg sample at 25 °C appear to be shifted with respect to the peak positions at 15 °C. From the lattice parameters calculated in Table 2, it can be seen that at α = 27 g CO_2_/kg sample the transition to hexagonal phase is completed, since the lattice parameters a of α = 27 and 38 g CO_2_/kg sample are the same.

At 15 °C, the lattice parameters at α = 27 and α = 38 g CO_2_/kg sample (Table 2) decrease slightly with increasing degree of CO_2_-loading, most likely due to the formation of more ordered aggregates at higher CO_2_ loading. On the other hand, at 25 °C, the lattice parameters of these two samples are the same, as mentioned above. However, the values at 25 °C are lower than the results obtained at 15 °C, probably due to higher mobility of the molecules. Nevertheless, it should be noted that the microstructures formed when α = 27 and 38 g CO_2_/kg sample are less defined than the ones of the unloaded sample, as it can be seen in Figure 3 and Figure 4. In general, loading with CO_2_ leads to peak-broadening, indicating that the distances between aggregates are less well-defined. In addition, the tails that the CO_2_-loaded samples present at low q values of the spectra suggest the presence of larger aggregates that could be of amorphous nature.

The change in the phase behavior of 60% L92/10% MEA is likely due to the increase in polarity of MEA during the loading process as a result of the formation of carbamate and ammonium ions by the reaction between MEA and CO_2_ [25]. This variation in the polarity increases the affinity of water (the most polar component in the system) for MEA, and at the same time decreases its affinity for the apolar polymer [14,15]. Increase in the solvent polarity favors the formation of normal structures over the reverse ones [11,12]. Therefore, one could infer that the hexagonal phases in the system are normal hexagonal phases, not reverse. In terms of packing parameter, the transition from lamellar to hexagonal phase can be explained by the presence of charged species embedded in the PEO blocks, which would increase the effective head group area, and therefore reduce the packing parameter. 

**Figure 5:**
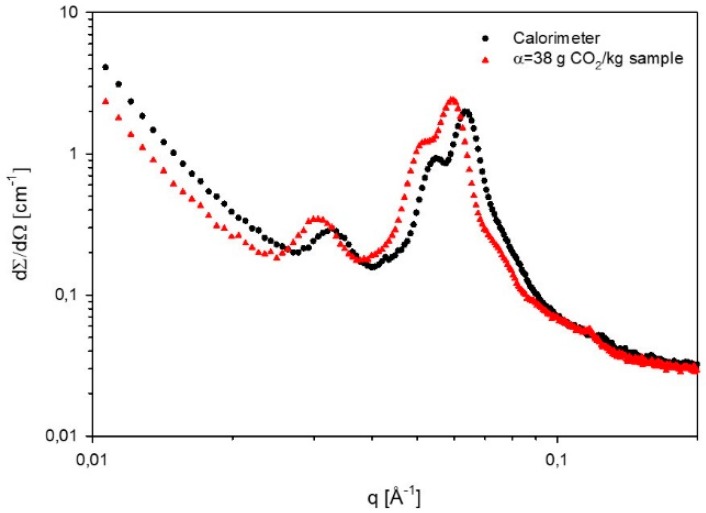
Comparison of the SAXS spectra of 60% L92/10% loaded in the calorimeter, and in the round bottom flask (α = 38 g CO_2_/kg sample).

The spectra of 60% L92/10% MEA with a loading of 38 g CO_2_/kg sample and the sample loaded in the calorimeter are compared in Figure 5. Both spectra follow the same pattern, and show the presence of hexagonal phases and larger aggregates. However, the peak positions are slightly shifted, which might be a result of slight differences in the sample composition. Therefore, the lattice parameter of the calorimeter-loaded sample is slightly larger than the value obtained for the sample with loading α = 38 g CO_2_/kg sample (Table 2). In general, when the polymer concentration decreases, the distance between aggregates (lattice parameters) increases [12].

### 3.3. Rheological Behavior

In order to understand the influence of CO_2_ uptake in the flowability, rheological properties of samples consisting of 60% L92/10% MEA with different degrees of CO_2_-loading were studied. The results shown are representative of all measurements performed using the same sample, and are reproducible within the same sample. 

#### 3.3.1. Viscosity

**Figure 6:**
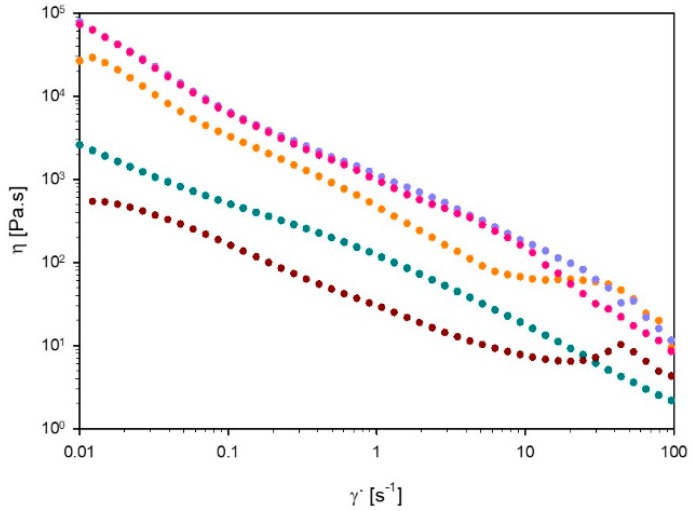
Viscosity (η) as a function of shear rate ($\dot{\gamma }$) of 60% L92/10% MEA α = 0 (⬤), α = 15 (⬤), α = 27 (⬤), and α = 38 g CO_2_/kg sample (⬤) at 25 °C, and α = 0 g CO_2_/kg sample (⬤) at 15 °C.

Figure 6 shows the viscosity of 60% L92/10% MEA at various degrees of CO_2_ loading, as a function of shear rate, as well as the viscosity of the unloaded sample at 15 °C. All samples exhibit shear thinning behavior. The moderate slope in viscosity versus shear rate precludes shear banding, for all samples. Shear thinning behavior is typical of samples consisting of large aggregates, such as lamellar and hexagonal microstructures [18]. With increasing shear rate, the aggregates disentangle, aligning with the flow direction and therefore decreasing the viscosity of the system [19]. Comparing the results of the unloaded sample at 15 and 25 °C, the viscosity of the sample increases with increasing temperature. Hexagonal phases are known to be more viscous than lamellar phases, which explains the increased viscosity of the sample formed by lamellar + hexagonal phases compared with the lamellar sample [35]. The viscosity of the unloaded sample is one order of magnitude lower than the viscosity of the other samples, throughout the investigated shear rate range. At a loading of 15 g CO_2_/kg sample, the viscosity at low shear rates is slightly lower than when the loading reaches 27 and 38 g CO_2_/kg sample. However, at ~60 s^−1^, the viscosity of the sample α = 15 g CO_2_/kg sample increases, and becomes approximately the same as the viscosity of the two remaining loaded samples. At loadings α = 27 and 38 g CO_2_/kg sample, the samples virtually show similar behavior, and their curves overlap at low shear rates. The viscosity difference between the samples with different degrees of loading can be explained by the change in the aggregates present in the samples. As the SAXS experiments show, the phase behavior of the sample changes as the reaction with CO_2_ proceeds. Moreover, the size of the aggregates also changes, as it is shown in Table 3. All these factors influence the overall viscosity of the sample. Samples with 27 and 38 g CO_2_/kg sample have almost identical viscosities because both samples have the same microstructure. The differences in behavior of these two samples can be a consequence of the slight change in composition, and the different degree of loading. Moreover, the high viscosity of the samples affected the loading time of the liquid crystals, which lasted over one week.

#### 3.3.2. Oscillatory Experiments

##### Strain Sweep

**Figure 7:**
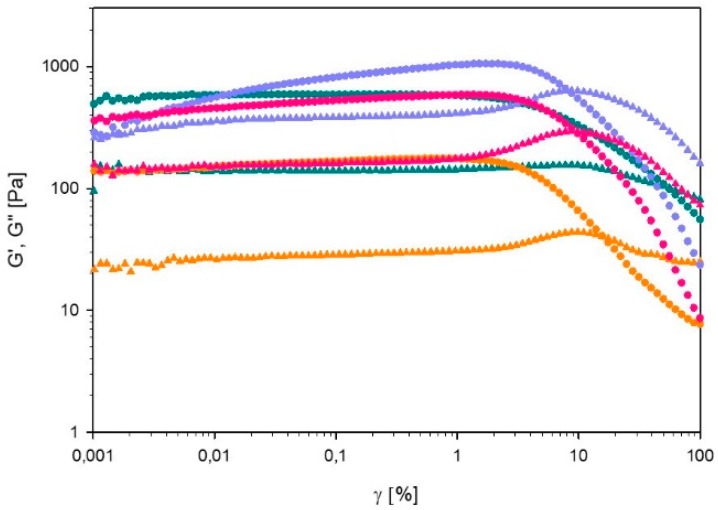
Storage modulus (G’) and loss modulus (G’’) of 60% L92/10% MEA α = 0 (⬤), α = 15 (⬤), α = 27 (⬤), and α = 38 g CO_2_/kg sample (⬤), as a function of strain amplitude (γ). G’ is depicted by full circles and G’’ by full triangles.

In Figure 7, G’ and G’’ of 60% L92/ 10% MEA with varying degrees of CO_2_ loading are plotted as a function of the strain amplitude. In all cases, G’ is higher than G’’ until the crossover point is reached. This point is found at 56.6% strain amplitude for the unloaded sample, and between 8–15% for the loaded ones. Overall, the addition of CO_2_ to the sample leads to a decrease of the gel strength, and changes the rheological behavior of the gel. The observed behavior could be explained in terms of the microstructure of the samples. According to the SAXS results, the unloaded sample has the most well-defined microstructure, therefore, the polymer molecules are more tightly packed, and more energy is required to disentangle the polymer molecules. For clarity, separate plots of G’ and G’’ are provided in the supplementary material.

In the case of α = 0, 15, and 38 g CO_2_/kg sample, G’ and G’’ are constant from low strain amplitudes to approximately 1%. Beyond this value, G’ starts to decrease. For α = 0, G’’ decreases slowly at higher strain amplitudes, which is consistent with type I LAOS behavior [20]. Beyond the linear regime, G’’ of α = 15, and 38 g CO_2_/kg sample first increases and starts decreasing after the crossover point, following type III behavior [20]. Type I behavior is described as strain thinning, where both G’ and G’’ decrease after the linear regime due to the disentanglement of polymer chains caused by increasing strain [20]. On the other hand, type III behavior is characterized by a weak strain overshoot (G’ decreases and G’’ first increases and then decreases) [20]. The explanation for type III behavior of hard gels provided by Hyun et al. can also be applied here [19]. Initially the molecules are arranged into large aggregates. At low strain amplitudes, G’ and G’’ are constant, but above a certain limit the connections between aggregates are broken. Therefore G’’ increases due to the breakage of the microstructures [16]. At higher strain amplitude, G’’ decreases again because the aggregates start sliding against each other in the direction of the flow. In the case of the sample with a loading of 27 g CO_2_/kg sample, G’ slowly increases until 1% strain is reached, and then decreases; whereas G’’ is constant until 1% strain and then it increases up to the crossover point, and subsequently it decreases. These trends are consistent with type IV behavior, which is caused by the strong interactions between molecules [36].

##### Frequency Sweep

**Figure 8:**
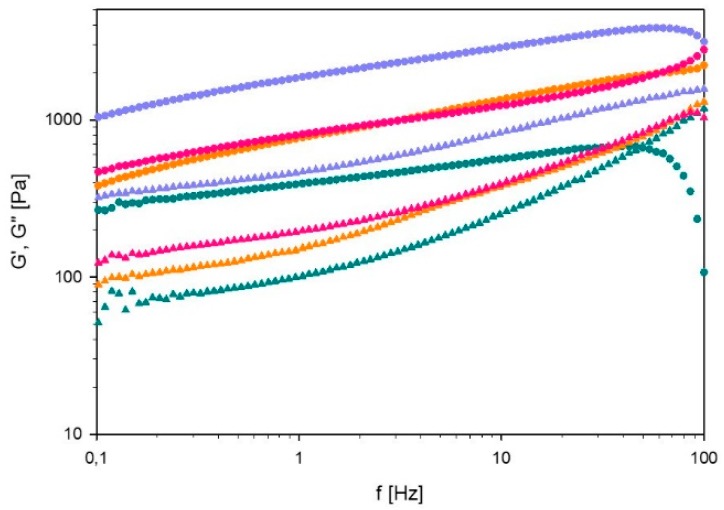
Storage modulus (G’) and loss modulus (G’’) of 60% L92/10% α = 0 (⬤), α = 15 (⬤), α = 27 (⬤), and α = 38 g CO_2_/kg sample (⬤), as a function of frequency (f). G’ is depicted by full circles and G’’ by full triangles.

The results of the frequency sweep of 60% L92/ 10% MEA with different degrees of CO_2_ loading are shown in Figure 8. The drop-off in G’ at high frequency is attributed to inertial artifacts [37]. The unloaded sample is the only one presenting a crossover between G’ and G’’ within the studied frequency range, which is found at 49.7 Hz. Therefore, this sample has the highest relaxation time, suggesting that the sample has a stronger viscous component than the CO_2_-loaded samples. Regarding CO_2_-loaded samples, G’ and G’’ increase with frequency but they do not cross below 100 Hz, indicating a more elastic material. Nevertheless, G’ is higher than G’’ for all the studied samples, suggesting that the samples have elastic behavior. For clarity, separate plots of G’ and G’’ are provided in the supplementary material. 

### 3.4. Thermal Degradation Experiments

**Figure 9:**
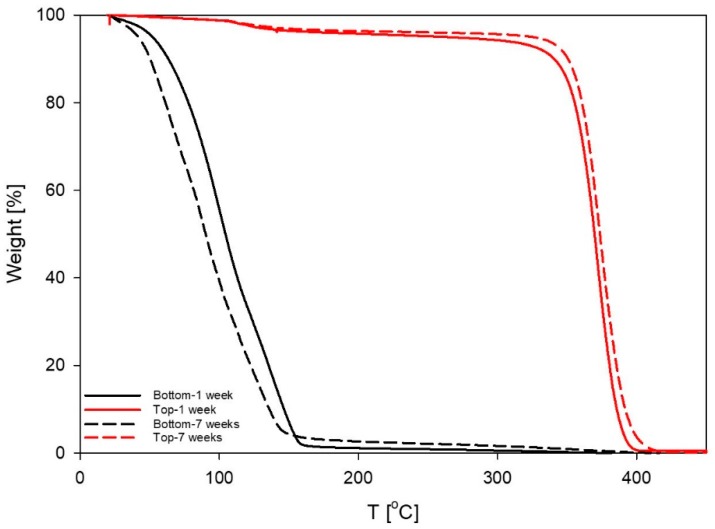
Thermogravimetric analysis (TGA) of the phases formed after storage of 60% L92/10% MEA at 80 °C for 1 and 7 weeks.

To study thermal degradation of MEA in the system, six parallels consisting of 60% L92/10% MEA with loading 38 g CO_2_/kg sample were placed into stainless steel cylinders and stored in a heating cabinet at 80 °C. Three parallels were stored for a week, and the remaining three samples were stored for 7 weeks [30]. After 1 and 7 weeks, all parallels had separated into two phases, but the weight of the samples remained constant. The two phases of one of the parallels of each set of samples were carefully separated and analyzed by TGA to determine the composition of each phase. The results showed that the upper phases contained approximately 96% of polymer, whereas the lower phases contained below 3% of polymer (Figure 9). Both phases were isotropic when examined by cross-polarized visual observation [8]. 

Amine analysis was used to determine the proportion of amine groups remaining in the samples before and after the thermal degradation experiments. Results are shown in Table 3. The sample with loading 38 g CO_2_/kg sample before degradation, and two samples from the thermal degradation experiments (after 1 week of heating, and after 7 weeks of heating) were titrated. For the samples of the thermal degradation experiments, the top and bottom phases were titrated separately to determine the amine content in each phase. The results show that there are no amine groups in the top phase of any of the two samples, indicating that it mostly consists of polymer, which is in agreement with the TGA results. On the other hand, the bottom phases after 1 and 7 weeks of storage contain 3.6 and 3.8 mol amine/(g CO_2_ + g sample), respectively, which is about three times more that the amine content of the un-degraded sample (α = 38 g CO_2_/kg sample). The un-degraded sample consisted of one phase and contained polymer, water and MEA. However, after heating the six parallels of this sample to 80 °C, the bottom phases only contain water and MEA, and therefore, the MEA concentration is higher than in the reference sample. If the overall amine concentration of the phase separated samples is estimated by calculating the ratio between the volume of both phases, the amine concentration is approximately the same as in the un-degraded sample. Therefore, the results suggest that after storage at 80 °C for 1 and 7 weeks, there was no actual thermal degradation of MEA. The estimated amine concentration after 7 weeks increases (3%) with respect to the original sample, which is within experimental error. 

Aqueous amine solutions are stable at 80 °C, and the results gained in this work indicate the L92 will not accelerate the MEA degradation, at least not at this low temperature [38]. In addition, the degradation experiments have also shown that the samples lose stability upon heating and phase separate, remaining in a two-phase state after being cooled down to room temperature. In a real process, phase separation would be beneficial because it would allow regeneration of the amine solvent separately from the polymer. Moreover, the formation of two isotropic phases would also be advantageous due to the lower viscosity of isotropic samples compared with liquid crystals. 

## 5. Conclusions

In this work, the behavior of the liquid crystalline sample 60% L92/10% MEA with different degrees of CO_2_-loading has been investigated. The maximum loading of 60% L92/10% MEA was 38.6 g CO_2_/kg sample when the pressure in the reactor reached 6 bar. The absorption capacity of the system is similar to that of aqueous 10% MEA. These results are slightly higher than the loading of thermotropic liquid systems reported in the literature. The phase behavior of samples with different degrees of CO_2_-loading was investigated by SAXS. As the degree of loading increases, the sample shifts from lamellar (15 °C) or coexisting lamellar and hexagonal phases (25 °C) toward hexagonal phases. The swelling of the samples increased with increasing degree of CO_2_-loading due to the higher polarity of the system, caused by the formation of carbamate and ammonium ions. In addition, the rheological behavior of the samples was studied. All samples showed shear thinning behavior. Regarding the oscillatory experiments, the unloaded sample presented LAOS type I behavior, whereas the remaining samples presented type III and type IV behaviors. Finally, the thermal stability of the sample with 38 g CO_2_/kg sample was investigated at 80 °C. At this temperature, there was no loss of amine groups due to degradation of MEA, indicating that the L92 does not favor the degradation of the amine. However, the samples phase separated into a top phase consisting of polymer, and a bottom phase consisting of MEA and water. Regarding the potential use of polymer/MEA/water liquid crystals for CO_2_ capture, transport and storage, one should take into account the strong changes in phase behavior and rheology in the system provoked by the formation of charged species. The high viscosity of the liquid crystals makes the loading of the samples slower than typical solvents for CO_2_ capture, and it is also disadvantageous for pumping and transport. Other compositions (or even other polymers) should be evaluated in order to obtain a liquid crystalline structure at the end of the loading process. However, this does not necessarily mean that the unloaded system needs to be liquid crystalline, as it has been shown here that the loading of CO_2_ into such a system can greatly affect the microstructure. This means that system design has to be carefully considered taking into account both the starting and end state. In addition, although the thermal stability of the loaded system is very low, the formation of two isotropic phases upon heating could be used as a process to unload the CO_2_ from the mixture after transport. 

## Figures and Tables

**Table 1:** Loading of 60% L92/10% MEA at different times.

Sample	CO_2_ Loading α (g CO_2_/kg Sample)
t = 1	14.9 ± 1.4
t = 2	22.6 *
t = 3	27.3 ± 0.3
t = 4	34.8 *
t = 5	37.7 ± 1.1

* The loadings of t = 2 and t = 4 were estimated from the correlation between the experimental data points.

**Table 2:** Lattice parameters of 60% L92/10% MEA at different times during reaction with CO_2_, and at different temperatures.

Sample	15 °C		25 °C	
	Phases	a, d [Å]	Phases	a, d [Å]
α = 0 g CO_2_/kg sample	Lα	123	Lα+H	a=173; d=126
α = 15 g CO_2_/kg sample	-	-	-	-
α = 27 g CO_2_/kg sample	H	278	H	237
α = 38 g CO_2_/kg sample	H	268	H	237
Calorimeter	-	-	H	243

**Table 3:** Amine content of 60% L92/10% MEA before loading, and after loading before and after storage at 80 °C.

Sample	Amine Groups (mol/g CO_2_ + g Sample)
α = 38 g CO_2_/kg sample	1.441 ± 0.002
Top phase (1 week)	N/A
Bottom phase (1 week)	3.628 ± 0.008
Top phase (7 weeks)	N/A
Bottom phase (7 weeks)	3.78 ± 0.010
Estimated degraded sample 1 week	1.425
Estimated degraded sample 7 weeks	1.485

## Supplementary Materials

The following are available online at http://www.mdpi.com/2073-4360/11/2/309/s1, Matlab code folder: matlab_code, Spreadsheet: GridCarbonResults.xls, Video: algorithm_movie.mp4.
