# Supplementary material for: CO2 in Lyotropic Liquid Crystals: Phase Equilibria Behavior and Rheology
Source: Polymers (Basel). 2019 Feb 12;11(2):309. doi: 10.3390/polym11020309 (PMC6419203; doi:10.3390/polym11020309)
Supplement: Supplementary file 1 [file polymers-11-00309-s001.pdf]

Supplementary Material

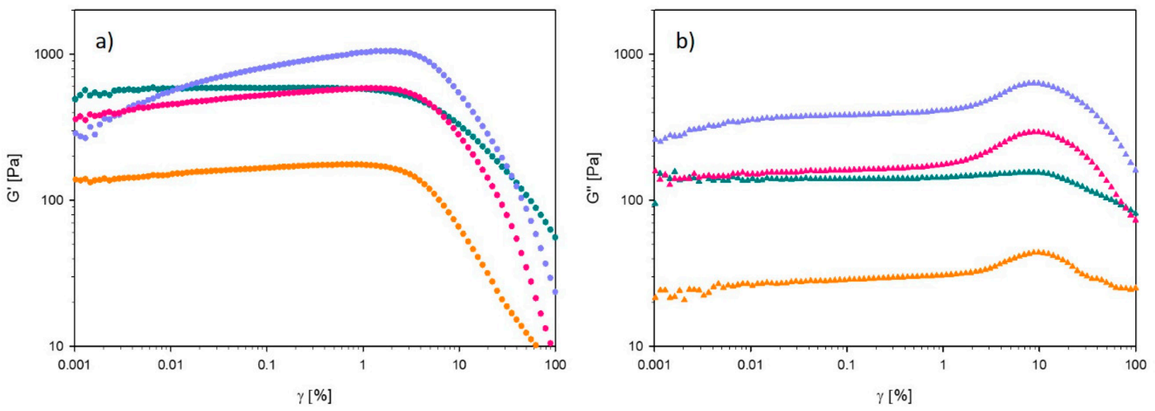

Figure 1. (a) Storage modulus (G’) and (b) loss modulus (G’’) of 60 % L92/10 % MEA α=0 (●), α=15 (●), α=27 (●), and α=38 g CO_2/kg sample (●), as a function of strain amplitude (γ).

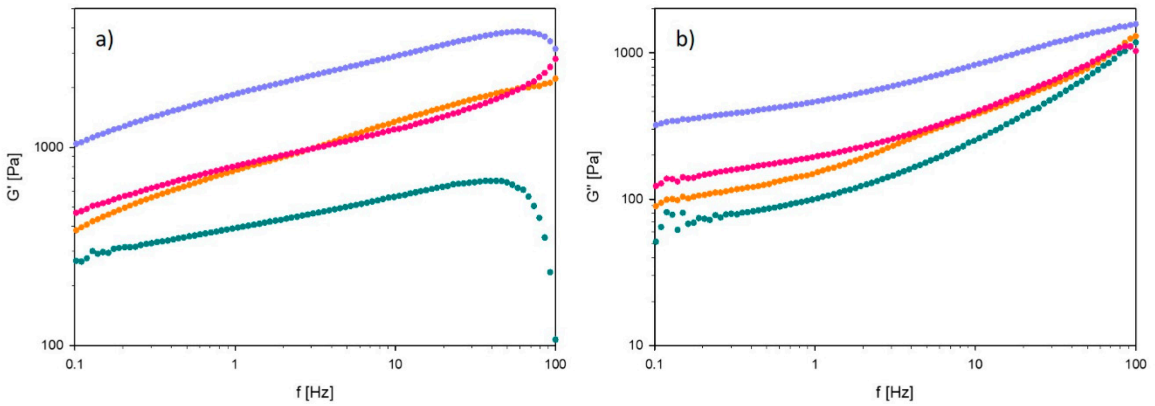

Figure 2. (a) Storage modulus (G’) and (b) loss modulus (G’’) of 60 % L92/10 % MEA α=0 (●), α=15 (●), α=27 (●), and α=38 g CO_2/kg sample (●), as a function of frequency (f).

Data for the loading of 10 % MEA obtained from Putta et al.[1]

α (g CO_2/kg sample)	P_{CO2} (bar)
7.21E-04	2.15E-09
7.28E-02	2.09E-07
1.45E-01	3.55E-07
2.17E-01	4.80E-07
2.89E-01	6.07E-07
3.61E-01	7.53E-07
4.33E-01	9.31E-07
5.05E-01	1.16E-06
5.77E-01	1.46E-06
6.49E-01	1.87E-06
7.21E-01	2.42E-06
7.93E-01	3.16E-06
8.65E-01	4.12E-06
9.37E-01	5.32E-06
1.01E+00	6.75E-06
1.08E+00	8.42E-06
1.15E+00	1.03E-05
1.23E+00	1.24E-05
1.30E+00	1.46E-05
1.37E+00	1.71E-05
1.44E+00	1.97E-05
1.51E+00	2.25E-05
1.59E+00	2.55E-05
1.66E+00	2.86E-05
1.73E+00	3.18E-05
1.80E+00	3.53E-05
1.87E+00	3.88E-05
1.95E+00	4.26E-05
2.02E+00	4.65E-05
2.09E+00	5.05E-05
2.16E+00	5.47E-05
2.23E+00	5.90E-05
2.31E+00	6.35E-05
2.38E+00	6.82E-05
2.45E+00	7.30E-05
2.52E+00	7.79E-05
2.59E+00	8.30E-05
2.67E+00	8.83E-05
2.74E+00	9.37E-05
2.81E+00	9.93E-05
2.88E+00	1.05E-04
2.95E+00	1.11E-04

3.03E+00	1.17E-04
3.10E+00	1.23E-04
3.17E+00	1.29E-04
3.24E+00	1.36E-04
3.32E+00	1.43E-04
3.39E+00	1.50E-04
3.46E+00	1.57E-04
3.53E+00	1.64E-04
3.60E+00	1.71E-04
3.68E+00	1.79E-04
3.75E+00	1.86E-04
3.82E+00	1.94E-04
3.89E+00	2.02E-04
3.96E+00	2.10E-04
4.04E+00	2.19E-04
4.11E+00	2.27E-04
4.18E+00	2.36E-04
4.25E+00	2.45E-04
4.32E+00	2.54E-04
4.40E+00	2.63E-04
4.47E+00	2.73E-04
4.54E+00	2.82E-04
4.61E+00	2.92E-04
4.68E+00	3.02E-04
4.76E+00	3.12E-04
4.83E+00	3.23E-04
4.90E+00	3.34E-04
4.97E+00	3.44E-04
5.04E+00	3.55E-04
5.12E+00	3.66E-04
5.19E+00	3.78E-04
5.26E+00	3.89E-04
5.33E+00	4.01E-04
5.40E+00	4.13E-04
5.48E+00	4.26E-04
5.55E+00	4.38E-04
5.62E+00	4.51E-04
5.69E+00	4.63E-04
5.76E+00	4.77E-04
5.84E+00	4.90E-04
5.91E+00	5.03E-04
5.98E+00	5.17E-04
6.05E+00	5.31E-04
6.13E+00	5.45E-04

6.20E+00	5.60E-04
6.27E+00	5.74E-04
6.34E+00	5.89E-04
6.41E+00	6.04E-04
6.49E+00	6.20E-04
6.56E+00	6.35E-04
6.63E+00	6.51E-04
6.70E+00	6.67E-04
6.77E+00	6.84E-04
6.85E+00	7.00E-04
6.92E+00	7.17E-04
6.99E+00	7.34E-04
7.06E+00	7.52E-04
7.13E+00	7.70E-04
7.21E+00	7.87E-04
7.28E+00	8.06E-04
7.35E+00	8.24E-04
7.42E+00	8.43E-04
7.49E+00	8.62E-04
7.57E+00	8.81E-04
7.64E+00	9.01E-04
7.71E+00	9.21E-04
7.78E+00	9.41E-04
7.85E+00	9.62E-04
7.93E+00	9.82E-04
8.00E+00	1.00E-03
8.07E+00	1.02E-03
8.14E+00	1.05E-03
8.21E+00	1.07E-03
8.29E+00	1.09E-03
8.36E+00	1.11E-03
8.43E+00	1.14E-03
8.50E+00	1.16E-03
8.58E+00	1.18E-03
8.65E+00	1.21E-03
8.72E+00	1.23E-03
8.79E+00	1.26E-03
8.86E+00	1.28E-03
8.94E+00	1.31E-03
9.01E+00	1.33E-03
9.08E+00	1.36E-03
9.15E+00	1.38E-03
9.22E+00	1.41E-03
9.30E+00	1.44E-03

9.37E+00	1.47E-03
9.44E+00	1.49E-03
9.51E+00	1.52E-03
9.58E+00	1.55E-03
9.66E+00	1.58E-03
9.73E+00	1.61E-03
9.80E+00	1.64E-03
9.87E+00	1.67E-03
9.94E+00	1.70E-03
1.00E+01	1.73E-03
1.01E+01	1.76E-03
1.02E+01	1.80E-03
1.02E+01	1.83E-03
1.03E+01	1.86E-03
1.04E+01	1.89E-03
1.04E+01	1.93E-03
1.05E+01	1.96E-03
1.06E+01	2.00E-03
1.07E+01	2.03E-03
1.07E+01	2.07E-03
1.08E+01	2.10E-03
1.09E+01	2.14E-03
1.10E+01	2.18E-03
1.10E+01	2.22E-03
1.11E+01	2.25E-03
1.12E+01	2.29E-03
1.12E+01	2.33E-03
1.13E+01	2.37E-03
1.14E+01	2.41E-03
1.15E+01	2.45E-03
1.15E+01	2.49E-03
1.16E+01	2.53E-03
1.17E+01	2.58E-03
1.17E+01	2.62E-03
1.18E+01	2.66E-03
1.19E+01	2.71E-03
1.20E+01	2.75E-03
1.20E+01	2.80E-03
1.21E+01	2.84E-03
1.22E+01	2.89E-03
1.22E+01	2.94E-03
1.23E+01	2.98E-03
1.24E+01	3.03E-03
1.25E+01	3.08E-03

1.25E+01	3.13E-03
1.26E+01	3.18E-03
1.27E+01	3.23E-03
1.28E+01	3.28E-03
1.28E+01	3.34E-03
1.29E+01	3.39E-03
1.30E+01	3.44E-03
1.30E+01	3.50E-03
1.31E+01	3.55E-03
1.32E+01	3.61E-03
1.33E+01	3.67E-03
1.33E+01	3.72E-03
1.34E+01	3.78E-03
1.35E+01	3.84E-03
1.35E+01	3.90E-03
1.36E+01	3.96E-03
1.37E+01	4.02E-03
1.38E+01	4.08E-03
1.38E+01	4.15E-03
1.39E+01	4.21E-03
1.40E+01	4.28E-03
1.41E+01	4.34E-03
1.41E+01	4.41E-03
1.42E+01	4.47E-03
1.43E+01	4.54E-03
1.43E+01	4.61E-03
1.44E+01	4.68E-03
1.45E+01	4.75E-03
1.46E+01	4.83E-03
1.46E+01	4.90E-03
1.47E+01	4.97E-03
1.48E+01	5.05E-03
1.48E+01	5.12E-03
1.49E+01	5.20E-03
1.50E+01	5.28E-03
1.51E+01	5.36E-03
1.51E+01	5.44E-03
1.52E+01	5.52E-03
1.53E+01	5.60E-03
1.53E+01	5.68E-03
1.54E+01	5.77E-03
1.55E+01	5.85E-03
1.56E+01	5.94E-03
1.56E+01	6.03E-03

1.57E+01	6.12E-03
1.58E+01	6.21E-03
1.59E+01	6.30E-03
1.59E+01	6.39E-03
1.60E+01	6.49E-03
1.61E+01	6.58E-03
1.61E+01	6.68E-03
1.62E+01	6.78E-03
1.63E+01	6.88E-03
1.64E+01	6.98E-03
1.64E+01	7.08E-03
1.65E+01	7.18E-03
1.66E+01	7.29E-03
1.66E+01	7.39E-03
1.67E+01	7.50E-03
1.68E+01	7.61E-03
1.69E+01	7.72E-03
1.69E+01	7.83E-03
1.70E+01	7.95E-03
1.71E+01	8.06E-03
1.71E+01	8.18E-03
1.72E+01	8.30E-03
1.73E+01	8.42E-03
1.74E+01	8.54E-03
1.74E+01	8.67E-03
1.75E+01	8.79E-03
1.76E+01	8.92E-03
1.77E+01	9.05E-03
1.77E+01	9.18E-03
1.78E+01	9.31E-03
1.79E+01	9.45E-03
1.79E+01	9.59E-03
1.80E+01	9.72E-03
1.81E+01	9.86E-03
1.82E+01	1.00E-02
1.82E+01	1.02E-02
1.83E+01	1.03E-02
1.84E+01	1.04E-02
1.84E+01	1.06E-02
1.85E+01	1.08E-02
1.86E+01	1.09E-02
1.87E+01	1.11E-02
1.87E+01	1.12E-02
1.88E+01	1.14E-02

1.89E+01	1.16E-02
1.90E+01	1.17E-02
1.90E+01	1.19E-02
1.91E+01	1.21E-02
1.92E+01	1.22E-02
1.92E+01	1.24E-02
1.93E+01	1.26E-02
1.94E+01	1.28E-02
1.95E+01	1.30E-02
1.95E+01	1.31E-02
1.96E+01	1.33E-02
1.97E+01	1.35E-02
1.97E+01	1.37E-02
1.98E+01	1.39E-02
1.99E+01	1.41E-02
2.00E+01	1.43E-02
2.00E+01	1.45E-02
2.01E+01	1.48E-02
2.02E+01	1.50E-02
2.02E+01	1.52E-02
2.03E+01	1.54E-02
2.04E+01	1.56E-02
2.05E+01	1.59E-02
2.05E+01	1.61E-02
2.06E+01	1.63E-02
2.07E+01	1.66E-02
2.08E+01	1.68E-02
2.08E+01	1.71E-02
2.09E+01	1.73E-02
2.10E+01	1.76E-02
2.10E+01	1.78E-02
2.11E+01	1.81E-02
2.12E+01	1.83E-02
2.13E+01	1.86E-02
2.13E+01	1.89E-02
2.14E+01	1.92E-02
2.15E+01	1.95E-02
2.15E+01	1.97E-02
2.16E+01	2.00E-02
2.17E+01	2.03E-02
2.18E+01	2.06E-02
2.18E+01	2.09E-02
2.19E+01	2.13E-02
2.20E+01	2.16E-02

2.20E+01	2.19E-02
2.21E+01	2.22E-02
2.22E+01	2.25E-02
2.23E+01	2.29E-02
2.23E+01	2.32E-02
2.24E+01	2.36E-02
2.25E+01	2.39E-02
2.26E+01	2.43E-02
2.26E+01	2.47E-02
2.27E+01	2.50E-02
2.28E+01	2.54E-02
2.28E+01	2.58E-02
2.29E+01	2.62E-02
2.30E+01	2.66E-02
2.31E+01	2.70E-02
2.31E+01	2.74E-02
2.32E+01	2.78E-02
2.33E+01	2.82E-02
2.33E+01	2.87E-02
2.34E+01	2.91E-02
2.35E+01	2.96E-02
2.36E+01	3.00E-02
2.36E+01	3.05E-02
2.37E+01	3.10E-02
2.38E+01	3.14E-02
2.39E+01	3.19E-02
2.39E+01	3.24E-02
2.40E+01	3.29E-02
2.41E+01	3.34E-02
2.41E+01	3.40E-02
2.42E+01	3.45E-02
2.43E+01	3.50E-02
2.44E+01	3.56E-02
2.44E+01	3.61E-02
2.45E+01	3.67E-02
2.46E+01	3.73E-02
2.46E+01	3.79E-02
2.47E+01	3.85E-02
2.48E+01	3.91E-02
2.49E+01	3.97E-02
2.49E+01	4.04E-02
2.50E+01	4.10E-02
2.51E+01	4.17E-02
2.51E+01	4.23E-02

2.52E+01	4.30E-02
2.53E+01	4.37E-02
2.54E+01	4.44E-02
2.54E+01	4.52E-02
2.55E+01	4.59E-02
2.56E+01	4.66E-02
2.57E+01	4.74E-02
2.57E+01	4.82E-02
2.58E+01	4.90E-02
2.59E+01	4.98E-02
2.59E+01	5.06E-02
2.60E+01	5.14E-02
2.61E+01	5.23E-02
2.62E+01	5.32E-02
2.62E+01	5.41E-02
2.63E+01	5.50E-02
2.64E+01	5.59E-02
2.64E+01	5.68E-02
2.65E+01	5.78E-02
2.66E+01	5.88E-02
2.67E+01	5.98E-02
2.67E+01	6.08E-02
2.68E+01	6.18E-02
2.69E+01	6.29E-02
2.69E+01	6.40E-02
2.70E+01	6.51E-02
2.71E+01	6.62E-02
2.72E+01	6.74E-02
2.72E+01	6.85E-02
2.73E+01	6.97E-02
2.74E+01	7.09E-02
2.75E+01	7.22E-02
2.75E+01	7.35E-02
2.76E+01	7.48E-02
2.77E+01	7.61E-02
2.77E+01	7.74E-02
2.78E+01	7.88E-02
2.79E+01	8.02E-02
2.80E+01	8.17E-02
2.80E+01	8.31E-02
2.81E+01	8.46E-02
2.82E+01	8.62E-02
2.82E+01	8.77E-02
2.83E+01	8.93E-02

2.84E+01	9.10E-02
2.85E+01	9.26E-02
2.85E+01	9.44E-02
2.86E+01	9.61E-02
2.87E+01	9.79E-02
2.87E+01	9.97E-02
2.88E+01	1.02E-01
2.89E+01	1.03E-01
2.90E+01	1.05E-01
2.90E+01	1.07E-01
2.91E+01	1.09E-01
2.92E+01	1.12E-01
2.93E+01	1.14E-01
2.93E+01	1.16E-01
2.94E+01	1.18E-01
2.95E+01	1.20E-01
2.95E+01	1.23E-01
2.96E+01	1.25E-01
2.97E+01	1.27E-01
2.98E+01	1.30E-01
2.98E+01	1.32E-01
2.99E+01	1.35E-01
3.00E+01	1.38E-01
3.00E+01	1.40E-01
3.01E+01	1.43E-01
3.02E+01	1.46E-01
3.03E+01	1.49E-01
3.03E+01	1.52E-01
3.04E+01	1.55E-01
3.05E+01	1.58E-01
3.06E+01	1.61E-01
3.06E+01	1.65E-01
3.07E+01	1.68E-01
3.08E+01	1.72E-01
3.08E+01	1.75E-01
3.09E+01	1.79E-01
3.10E+01	1.82E-01
3.11E+01	1.86E-01
3.11E+01	1.90E-01
3.12E+01	1.94E-01
3.13E+01	1.98E-01
3.13E+01	2.02E-01
3.14E+01	2.07E-01
3.15E+01	2.11E-01

3.16E+01	2.15E-01
3.16E+01	2.20E-01
3.17E+01	2.25E-01
3.18E+01	2.30E-01
3.18E+01	2.35E-01
3.19E+01	2.40E-01
3.20E+01	2.45E-01
3.21E+01	2.50E-01
3.21E+01	2.56E-01
3.22E+01	2.61E-01
3.23E+01	2.67E-01
3.24E+01	2.73E-01
3.24E+01	2.79E-01
3.25E+01	2.85E-01
3.26E+01	2.91E-01
3.26E+01	2.98E-01
3.27E+01	3.05E-01
3.28E+01	3.12E-01
3.29E+01	3.19E-01
3.29E+01	3.26E-01
3.30E+01	3.33E-01
3.31E+01	3.41E-01
3.31E+01	3.48E-01
3.32E+01	3.56E-01
3.33E+01	3.65E-01
3.34E+01	3.73E-01
3.34E+01	3.82E-01
3.35E+01	3.90E-01
3.36E+01	3.99E-01
3.36E+01	4.09E-01
3.37E+01	4.18E-01
3.38E+01	4.28E-01
3.39E+01	4.38E-01
3.39E+01	4.48E-01
3.40E+01	4.59E-01
3.41E+01	4.70E-01
3.42E+01	4.81E-01
3.42E+01	4.92E-01
3.43E+01	5.04E-01
3.44E+01	5.16E-01
3.44E+01	5.28E-01
3.45E+01	5.40E-01
3.46E+01	5.53E-01
3.47E+01	5.66E-01

3.47E+01	5.80E-01
3.48E+01	5.94E-01
3.49E+01	6.08E-01
3.49E+01	6.23E-01
3.50E+01	6.38E-01
3.51E+01	6.53E-01
3.52E+01	6.69E-01
3.52E+01	6.85E-01
3.53E+01	7.01E-01
3.54E+01	7.18E-01
3.55E+01	7.35E-01
3.55E+01	7.53E-01
3.56E+01	7.71E-01
3.57E+01	7.90E-01
3.57E+01	8.09E-01
3.58E+01	8.29E-01
3.59E+01	8.49E-01
3.60E+01	8.69E-01
3.60E+01	8.90E-01
3.61E+01	9.12E-01
3.62E+01	9.33E-01
3.62E+01	9.56E-01
3.63E+01	9.79E-01
3.64E+01	1.00E+00
3.65E+01	1.03E+00
3.65E+01	1.05E+00
3.66E+01	1.08E+00
3.67E+01	1.10E+00
3.67E+01	1.13E+00
3.68E+01	1.16E+00
3.69E+01	1.18E+00
3.70E+01	1.21E+00
3.70E+01	1.24E+00
3.71E+01	1.27E+00
3.72E+01	1.30E+00
3.73E+01	1.33E+00
3.73E+01	1.36E+00
3.74E+01	1.40E+00
3.75E+01	1.43E+00
3.75E+01	1.46E+00
3.76E+01	1.50E+00
3.77E+01	1.53E+00
3.78E+01	1.57E+00
3.78E+01	1.60E+00

3.79E+01	1.64E+00
3.80E+01	1.68E+00
3.80E+01	1.72E+00
3.81E+01	1.76E+00
3.82E+01	1.80E+00
3.83E+01	1.84E+00
3.83E+01	1.88E+00
3.84E+01	1.93E+00
3.85E+01	1.97E+00
3.85E+01	2.02E+00
3.86E+01	2.06E+00
3.87E+01	2.11E+00
3.88E+01	2.16E+00
3.88E+01	2.21E+00
3.89E+01	2.26E+00
3.90E+01	2.31E+00
3.91E+01	2.36E+00
3.91E+01	2.41E+00
3.92E+01	2.46E+00
3.93E+01	2.52E+00
3.93E+01	2.57E+00
3.94E+01	2.63E+00
3.95E+01	2.69E+00
3.96E+01	2.75E+00
3.96E+01	2.81E+00
3.97E+01	2.87E+00
3.98E+01	2.93E+00
3.98E+01	2.99E+00
3.99E+01	3.05E+00
4.00E+01	3.12E+00
4.01E+01	3.19E+00
4.01E+01	3.25E+00
4.02E+01	3.32E+00
4.03E+01	3.39E+00
4.04E+01	3.46E+00
4.04E+01	3.54E+00
4.05E+01	3.61E+00
4.06E+01	3.68E+00
4.06E+01	3.76E+00
4.07E+01	3.84E+00
4.08E+01	3.92E+00
4.09E+01	3.99E+00
4.09E+01	4.08E+00
4.10E+01	4.16E+00

4.11E+01	4.24E+00
4.11E+01	4.33E+00
4.12E+01	4.41E+00
4.13E+01	4.50E+00
4.14E+01	4.59E+00
4.14E+01	4.68E+00
4.15E+01	4.77E+00
4.16E+01	4.87E+00
4.16E+01	4.96E+00
4.17E+01	5.06E+00
4.18E+01	5.15E+00
4.19E+01	5.25E+00
4.19E+01	5.35E+00
4.20E+01	5.46E+00
4.21E+01	5.56E+00
4.22E+01	5.66E+00
4.22E+01	5.77E+00
4.23E+01	5.88E+00
4.24E+01	5.99E+00
4.24E+01	6.10E+00

References

1. Putta, K. R.; Pinto, D. D. D.; Svendsen, H. F.; Knuutila, H. K., CO_2 Absorption into Loaded Aqueous Mea Solutions: Kinetics Assessment Using Penetration Theory. *International Journal of Greenhouse Gas Control* **2016,** *53*, 338-353, https://doi.org/10.1016/j.ijggc.2016.08.009.
